# A Review of Data Analytic Applications in Road Traffic Safety. Part 2: Prescriptive Modeling

**DOI:** 10.3390/s20041096

**Published:** 2020-02-17

**Authors:** Qiong Hu, Miao Cai, Nasrin Mohabbati-Kalejahi, Amir Mehdizadeh, Mohammad Ali Alamdar Yazdi, Alexander Vinel, Steven E. Rigdon, Karen C. Davis, Fadel M. Megahed

**Affiliations:** 1 Department of Industrial and Systems Engineering, Auburn University, Auburn, AL 36849, USA; qzh0011@auburn.edu (Q.H.); azm0127@auburn.edu (A.M.); 2 College for Public Health and Social Justice, Saint Louis University, St. Louis, MO 63103, USA; miao.cai@slu.edu (M.C.); steve.rigdon@slu.edu (S.E.R.); 3 Jack H. Brown College of Business and Public Administration, California State University at San Bernardino, San Bernardino, CA 92407, USA; nasrin.mohabbati@csusb.edu; 4 Johns Hopkins Carey Business School, Baltimore, MD 21202, USA; yazdi@jhu.edu; 5 Department of Computer Science and Software Engineering, Miami University, Oxford, OH 45056, USA; karen.davis@miamioh.edu; 6 Farmer School of Business, Miami University, Oxford, OH 45056, USA

**Keywords:** crash risk modeling, hazardous materials, highway safety, operations research, prescriptive analytics, shortest path problem, trucking, vehicle routing problem

## Abstract

In the first part of the review, we observed that there exists a significant gap between the predictive and prescriptive models pertaining to crash risk prediction and minimization, respectively. In this part, we review and categorize the optimization/ prescriptive analytic models that focus on minimizing crash risk. Although the majority of works in this segment of the literature are related to the hazardous materials (hazmat) trucking problems, we show that (with some exceptions) many can also be utilized in non-hazmat scenarios. In an effort to highlight the effect of crash risk prediction model on the accumulated risk obtained from the prescriptive model, we present a simulated example where we utilize four risk indicators (obtained from logistic regression, Poisson regression, XGBoost, and neural network) in the *k*-shortest path algorithm. From our example, we demonstrate two major designed takeaways: (a) the shortest path may not always result in the lowest crash risk, and (b) a similarity in overall predictive performance may not always translate to similar outcomes from the prescriptive models. Based on the review and example, we highlight several avenues for future research.

## 1. Introduction

In Part 1 of this review, we have discussed in detail how information extracted from different sensing technologies is utilized in explaining and predicting motor vehicle crash risk as a function of internal/driver-related factors (i.e., fatigue and distracted driving) and external conditions (i.e., weather, traffic, and road geometry characteristics), focusing on the first half of the data analytics modeling procedure (i.e., data collection ⟶ data exploration ⟶ predictive modeling). In Part 2, we discuss how this information is incorporated in prescriptive routing models, where crash risk is controlled/minimized through route/path selection or rest-break scheduling. The reader should note that these optimization-based route selection models do not reflect all possible approaches to translate the outputs from Part 1 into actions that improve motor vehicle safety. For example, we do not review regulatory/policy-related intervention studies and motor vehicle safety technologies (e.g., anti-lock braking systems, lane departure detection systems, and collision avoidance technologies) as the scope/length of this review would greatly increase. Instead, we focus on the routing/path selection prescriptive models since they attempt to account for the “human-in-the-loop” aspect of driving. Specifically, such studies examine how to make “safer” decisions with regard to routing/path selection, while utilizing sensing technologies to account for weather, traffic, and/or road-geometric conditions.

Routing studies are primarily geared toward trucking operations, especially hazardous materials (hazmat), as truck drivers have a unique working environment, where (a) they encounter different routes/paths, weather, traffic conditions, and locations each time they take a trip; (b) they are on the road for long hours with little supervision or contact with fellow employees [1]; (c) they can be driving for varying lengths of time since they are affected by the scheduling requirements of the motor carrier, shipper, and receiver [2]; and (d) the driver’s sleep quality/duration is often negatively affected by the working environment [2]. These characteristics of truck drivers’ environment can increase their cognitive demands and/or fatigue rates when compared to other drivers [3]. For this reason, large trucks are often involved in the most severe crashes. In the U.S., “large trucks and buses account for 12% of the traffic fatalities” [4], while accounting for only 9% of the total miles driven in the U.S. [5]. Furthermore, in the case of hazmat trucking, the severity of the crash can be dramatically increased with the spread/leakage of hazardous materials that can be dispersed to a much larger area, affecting humans, animals, and/or plants that were not in the immediate vicinity of the crash. Consequently, hazmat trucking operators are especially incentivized to explicitly account for traffic incident risks. This, in turn, explains why a majority of motor vehicle routing studies that include safety component are concerned with hazmat operators.

Despite the large body of research on how to improve motor vehicle safety in both the predictive and prescriptive modeling fields, studies combining outcomes from advanced explanatory/predictive models as inputs to the optimization/routing models are few, as we highlight throughout this paper. From a practical perspective, this is problematic since the effectiveness of any model is greatly impacted by the quality of its inputs. Therefore, while categorizing prescriptive data analytics approaches to motor vehicle safety research, our goal is to explicitly highlight the inputs/assumptions in these models in an effort to outline pathways for incorporating the most recent and precise predictive results. Note also, that although hazmat transportation is the most widely used application for prescriptive models surveyed, insofar as they reduce the overall incident risk, such models (or at least their generalizations) often can be directly applied to general trucking operations or even personal vehicles. Finally, to make the analysis more applicable, we also present a stylized case study, which, while based on simulated data, illustrates some issues and difficulties in combining the two streams of research.

The remainder of the second part of this review is organized as follows. We provide some necessary background on hazmat trucking operations in Section 2. Then, in Section 3, we categorize the optimization models used for minimizing the risk of hazmat truck crashes and/or their severity based on three different perspectives: (a) risk models used; (b) types of decision variables, input parameters, objective function(s), and constraints; and (c) types of algorithms and computational methods utilized. To illustrate the effect of different crash risk predictive models on the prescriptive model’s outcome, we provide an illustrative example in Section 4. Our concluding remarks are presented in Section 5. Similar to Part 1, we also provide Supplementary Materials, where we present a link to an *R Markdown* notebook containing our illustrative example’s code and analysis.

## 2. Background: Hazmat Trucking Operations

According to the Pipeline and Hazardous Material Safety Administration (PHMSA) of the US Department of Transportation (USDOT), a hazmat is defined as any substance or material that is toxic, explosive, corrosive, combustible, poisonous, or radioactive that is capable of becoming a threat to the environment, properties, and people’s safety [6]. Hazardous materials are divided into the following nine categories: (a) explosives, (b) gases, (c) flammable liquids, (d) flammable solids, (e) oxidizers and organic peroxides, (f) toxic materials and infectious substances, (g) radioactive materials, (h) corrosive materials, and (i) miscellaneous dangerous goods [6].

The most important difference between hazmat and non-hazmat transportation is that moving hazmat raises an inherent risk for public safety and environment. A hazmat incident can occur in origin during loading, in transit, in transit storage, and in destination during unloading [7]. Even though hazmat incidents are not common, their occurrence leads to catastrophic consequences such as fatalities, severe injuries, and property/environmental damages. In 2018, 19,581 incidents including explosions, fires, and poisonous gas leakage were reported in the U.S. These incidents caused four fatalities, 127 injuries, $80 million property damage, and a huge effort of evacuating and restoring the affected areas [7]. Most of the fatalities and damages occurred on highways (approximately 90% of the reported incidents in 2018 [7]), emphasizing the importance of in-land hazmat transportation planning and routing. For this reason, most of the papers in the literature studied hazmat route planning on highways and roads; hereafter, we only consider such applications.

Hazmat transportation planning has traditionally received the attention of both carriers and regulators. Carriers tend to plan each shipment separately with the goal of minimizing travel time/cost, while complying with any regulations and risk management considerations. On the other hand, regulators consider all the shipments in the road network and work on promoting risk equity through various network design measures [8].

Hazardous materials (hazmat) routing problems can be categorized into two classes based on the different perspectives of the parties involved. The simplest type of the hazmat transportation routing problem deals with an origin and a destination (an O–D pair) and one type of hazmat to be shipped on a given road network. Thus, a single route will be chosen as the optimal solution for the problem with the objective of minimizing both the cost and risk. This class of the problems with a single O–D pair and single shipment is usually referred to as *local route planning*. In this type of problem, each shipment is planned separately, not taking into account all other shipments. A more general version, involving several O–D pairs with several shipments, can still be referred to as local as long as each is treated separately from the point of view of transportation risk analysis. On the other hand, it is often observed that such an approach can lead to overloaded hazmat traffic on certain links of the network, leading to increase in incident probabilities and risk inequity. If multiple commodities are shipped through multiple routes with the objective of minimizing cost and risk as well as promoting risk equity among all regions, then such problems are usually addressed as *global routing planning*. Some examples of problems modeled from the operational point of view (i.e., local route planning) can be found in [9,10,11,12,13,14,15,16,17,18,19,20]. On the other hand for the network design perspective (i.e., global route planning), the reader is referred to the examples in [21,22,23,24,25,26,27,28].

Both problems have been extensively studied in the literature. Note though, that as far as applications to general motor vehicles are concerned, the global hazmat transportation problem is not particularly transferable, as the primary reason to consider a risk equity criterion is related to considerable change in risk exposure of the communities due to relatively heavy hazmat traffic. Furthermore, for obvious reasons, we will also not consider the policy-making literature discussing important decisions such as (a) road segments closure [29], (b) toll-setting [30,31,32,33], (c) locating waste treatment centers in safe sites [34,35,36,37,38], (d) locating hazmat emergency response teams [39,40,41], etc.

## 3. Optimization Models for Minimizing Crash Risks/Costs

In this section, we review optimization models used for the prescriptive component of crash risk analysis. As noted above, the vast majority of relevant literature originates in the area of hazmat transportation. The potential for extremely impactful incidents means that risk consideration is a primary criterion in decision making for routing of such vehicles, which leads to a wide section of the literature dedicated to vehicle routing problems (VRPs) for hazmat transportation. Consequently, any analysis of general purpose safety-enabled routing has to rely on the extensive existing developments in hazmat literature. Therefore, for the discussion in this section, we first consider the topic of risk models used in hazmat transportation. This classification of the literature will let us identify aspects of models and corresponding approaches that can be applied more widely to general motor vehicles. We then focus on characterising the existing optimization literature according to (a) model type based on how the underlying parameters are treated, (b) basic optimization model elements (variables, objective function and constraints), and (c) the type of algorithmic approaches used.

### 3.1. Risk Models in Hazmat Transportation

To incorporate a stochastic parameter (e.g., traffic incidents) into a prescriptive model, it is not enough to determine the probability of an incident on each arc. One also needs to select a way to quantitatively measure and compare the risks associated with potential alternative decisions. In the case of hazmat transportation, Batta and Kwon (2013) [8] identified the following three important building blocks for risk measurement: (a) *incident probability*, (b) *exposed population*, and (c) *expected consequence*. Intuitively, the incident probability focuses on measuring the probability of an undesirable event, while the exposed population refers to the measure of potential effect. Either can be used in its own right. For example, if the incident probability is constant, then the exposed population can be employed as the primary way to differentiate between decisions [14,34,42]. Alternatively, if it is impossible to adequately estimate the potential effects, then incident probability can be used on its own [43]. However, when we can estimate both measures, combining both of them through the *expected consequence* measure allows for a more complete picture. *Expected consequence* is defined as the expected value for the at-risk population taking into account the incident probability along the selected route. Note that other risk indicators have been proposed and used in the literature. These risk indicators present different penalization functions and focuses when compared to the traditional three measures. We present an overview of the indicators and the papers utilizing these approaches in hazmat settings in Table 1.

A number of factors must be taken into account when picking a specific risk indicator. First, there is not a model that is strictly superior to all others. Second, it can be seen from the formulations presented in Table 1 that these indicators have different objectives and assumptions. For example, the traditional *expected consequence* approach assumes a risk-neutral preference. On the other hand, the perceived risk, value at risk and conditional value at risk all introduce risk-averse decision making criteria. Specifically, the perceived risk model introduces a risk parameter *k* involved in higher-moment “perceived” loss evaluation [50,51]. As the concept of minimizing risk is not inherent to transportation problems, a detailed discussion of the properties of these methods can be obtained from the general stochastic optimization literature (see, e.g., [19,20,25,52,56]). Third, in the case of non-hazmat problems, incidence consequence is typically not a major decision factor as the consequences are primarily related to speed and the number of vehicles involved in a crash. These consequences are typically hard to estimate beforehand and thus, the use of crash probabilities is often the preferred approach.

Most of the cited literature in Table 1 operates in a static fashion. Specifically, most papers assume a constant hazmat accident rate (usually between $10^{-8}$–$10^{-6}$ per vehicle-mile), which is based on the work of [10]. However, crash risk is affected by weather/traffic among other conditions. These parameters tend to be time-variant, and thus a constant probability does not account for the findings in the crash risk prediction modeling domain. We recommend that the optimization literature should focus on more dynamic conditions to account for the time-varying factors affecting crash risk. It is important to note that most of the existing risk indicators, such as the ones shown in Table 1, can account for time-varying conditions. For example, Toumazis and Kwon [19] showed that CVaR-based models can be used for dynamic models, where the risk and cost are time-dependent. Therefore, it can be used with the more advanced statistical models discussed in Section 5 of Part 1.

Based on the discussion in this subsection, one can see that hazmat risk models typically consider/emphasize the consequences/severity of a crash when it happens. For non-hazmat vehicles, the severity of a crash would depend on the number/type of vehicles involved, the type of collision, speed differential, etc. Although these are also true in hazmat case, the literature typically considers the “worst case outcome”, where the probability of dispersion is utilized to capture the consequences of hazmat releases. Thus, in such cases, the effect on the involved vehicles is often ignored since it is assumed to be minor when compared to the health-outcomes and cleaning efforts that are associated with containing hazmat materials. On the other hand, the severity of non-hazmat crashes is dependent on (a) the potential for injuries/fatalities and (b) the traffic buildups seen by other commuters. Given that these two factors are relatively hard to predict/model for non-hazmat crashes, reducing the likelihood of a crash represents the important component of risk models for non-hazmat vehicles. Consequently, this component should be reflected in the choice of an appropriate risk model.

### 3.2. Classification Based on Model Type

In this section, we classify the relevant transportation (hazmat) optimization papers based on the underlying parameters. Our classification combines the taxonomies presented in Erkut et al. [57] and Pradhananga et al. [47]. Erkut et al. [57] differentiated hazmat transportation models based on whether the proposed solution will update in time according to new information. Their approach divided the literature into (a) a priori optimization, where model updating is not permitted; (b) adaptive-route selection, if the result will be updated subject to the realization of certain data; and (c) adaptive route selection in real-time, if the updating considers real-time changes in the data. On the other hand, Pradhananga et al. [47] divided the literature according to (a) deterministic/static and (b) stochastic/dynamic models. Thus, by combining both classifications, we obtain six categories. The definition of each group (G) and a sample of its literature are presented in Table 2.

Based on Table 2, there are several observations to be made. First, we classified most of the papers that include some version of dynamic parameters as semi-dynamic (see, e.g., Group 4). Our rationale for this classification is that these papers do not provide any discussion on updating the solution en route. Second, the existence of semi-dynamic or truly-dynamic parameters does not mean that these papers should be considered as such in non-hazmat applications. For example, in [42], the dynamic parameters correspond to evaluation of incident consequences for hazmat transportation (e.g., real-time population within the affected area). Although this allows us to classify them as semi-dynamic, these parameters are irrelevant for general transportation routing applications. Third, the limited research in Groups 5–6 shows that there is an opportunity to capitalize on the availability of real-time information of important inputs to improve the mathematical models’ performances in practice (as shown in the results of Qu et al. [67]). Fourth, extending the models in Group 4 (or 3) to Group 6 (or 5) models can be achieved with relative ease through providing (a) a procedure for periodic real-time update of the underlying parameters and (b) well-defined criteria for periodic re-optimization. A case in point is the model presented in Kang et al. [20]. The problem there is solved with a two-stage solution procedure based on either Dijkstra’s method or a heuristic. For a practical case with 90 intersections and 148 road segments the solution time does not exceed five seconds. Therefore, with a clear criterion for updating the solution (e.g., every 10 min, or whenever significant change in risk estimation is observed), it can be efficiently adapted to a truly dynamic model.

One additional benefit from categorizing the optimization models based on the risk model is that it can help us better understand some of the inherent limitations/assumptions of the optimization model. For example, based on Part 1, *traffic* and *weather* conditions were found to be important risk factors in many models. As these conditions can vary dramatically over the course of the drive, the truly-dynamic and stochastic optimization models would be a better choice in most applications since they can capture the time-varying nature of the inputs.

### 3.3. Classification Based on the Types of Decision Variables, Input Parameters, Objective Function(s), and Constraints

#### 3.3.1. Type of Decision Variables

In many trucking safety problems, binary decision variables are used to define the type of decisions to be achieved by optimizing a particular model. The models can be divided based on whether the variables reflect decisions made on arc or path level. For example, in the context of a single O–D routing problem and an-arc based formulation, a value of 1 indicates that the driver should be routed through this arc, and 0 otherwise. More generally, if there are multiple trucks in the system, the decision can represent whether a certain truck should deliver a product for a given customer using a given arc. To illustrate this concept, let us consider the notation used in [64], where the binary variable $x_{ijv}^{\tau }$ is set to one, whenever truck *v* is leaving node *i* at specific time τ by using the link (i,j). On the other hand, one may define variables that are indexed over whole paths rather than separate arcs (see [56], for an example of such a formulation). If such an approach is followed, practitioners are required to pre-compute a number of candidate paths between all O–D pairs in advance. This approach can be particularly useful when attempting a real-time update of the solution, as it can significantly reduce the computational effort required. Concurrently, it creates a separate problem of selecting a set of pre-computed paths, which, if done poorly, can limit the quality of the realized solutions. This means that there is a trade-off between both methods, and their pros and cons should be considered prior to model construction.

#### 3.3.2. Types of Input Parameters

Depending on the assumptions of the model, availability of data, and application, the inputs to the prescriptive models can differ significantly. In the context of attempting to minimize crash risk, different types of parameters can correspond to different sources of risk, as well as different system components that can affect this risk. In addition, most of the problems also include various parameters generally associated with vehicle routing problems, e.g., time windows, vehicle parameters, etc. Based on our review, we identified 11 types of parameters used in the literature. Table 3 provides a brief description of each type along with citations for when each type was used. Note that these parameters are not mutually exclusive, and thus several of the papers can be found at different rows within the table. Additionally, for some of these types (e.g., traffic flows, road/weather conditions, and/or exposed populations), it may be important to consider real-time updates. Models using those parameters can, in principle, capitalize on the advanced statistical models highlighted in the explanatory/predictive modeling section. Hereafter, we use the type ID (i.e., the number) to refer to a specific parameter type.

From Table 3, it should be apparent that most optimization models do not include parameters that relate to traffic, weather, and road geometric conditions. Although this should not be a surprising observation based on the bibliometric analysis performed in Part 1, it is a potentially problematic observation since at least one of those factor sets was deemed important by most of the explanatory/predictive modeling studies reviewed in Part 1. As a consequence, we estimate that crash risk would be underestimated by the optimization models in the case of adverse weather, traffic and road conditions. This is an important gap in the prescriptive modeling literature that needs to be further investigated.

#### 3.3.3. Type of Objective Functions used in Hazmat Transportation

There are two main objectives in crash risk optimization models: economic savings and minimizing the total risk. Economic savings relates to improving travel time, distance, and other corresponding costs. Total risk represents the economic or other type of loss associated with transportation incidents. Usually, the total risk is evaluated as a cumulative effect over the selected route. Furthermore, it is typical to assume that incident occurrence along each arc is independent, which in conjunction with very small incident probabilities leads to the standard assumption that the total probability along a route can be estimated through summing the probabilities on each arc. Note that the two objectives are not necessarily conflicting since it is not always the case that shorter routes are more risky.

There are two general ways to address multiple objectives in optimization models: (a) using a weighted sum method to get a single linear objective function (see, e.g., [40,45,60,64,65]) or (b) keep the multiple objectives and find a set of non-dominated solutions (as in [14,46,47,51]). Sometimes, it may be possible to introduce a natural problem-specific way to combine the objectives. For example, in [65], the objective in the model is to minimize both travel cost and risk, but the authors present a way to integrate the direct freight cost as a component related to risk which is decided by the frequency and leakage probability. From a solution perspective, a key disadvantage of merging multiple objectives into one function (by using a generic weighed sum method) is that it is often difficult to find satisfactory weights, and the result will be sensitive to the weight assigned. On the other hand, methods that aim at generating the full efficient frontier often require significant computational effort, especially if the underlying single-objective relaxation is hard to solve on its own.

In Table 4, we categorize the surveyed papers in this section according to the type of objective used (while integrating the information of parameters by applying the type ID from Table 3). From the table, one can observe the following, (a) most papers have focused on minimizing risk instead of a purely economic model, and (b) most papers attempt to optimize multiple objectives. In addition, with the exception of [65], the papers incorporated only two to three parameter types. In our view, the limited number of parameter types considered in the optimization model (despite the different objectives) reflects the divide between the crash risk prediction modeling and optimization literatures. For example, traffic conditions (PT-ID 5) and weather conditions (PT-ID 6) were considered twice and once, respectively. However, they are important crash risk predictors as shown in the references cited in the explanatory/predictive modeling section.

#### 3.3.4. Structure of Constraints in Hazmat Transportation

Similar to the previous subsections, the constraints that are widely used in optimization models can be grouped into two families: general vehicle routing constraints and those related to evaluation of risk. The general VRP constraints are well understood in the literature, and are enforced to make sure that the proposed transportation plan is feasible, i.e., loading capacity is not exceeded, the demand is satisfied, delivery time windows are observed, etc. [40,64]. Risk-specific constraints, on the other hand, are closely related to the objectives; it is often possible to consider a risk term as an objective or a constraint depending on whether the decision maker is interested in achieving a minimal risk, or satisfying a risk threshold. Some model-specific constraints can also be used; for example, in [25], the authors consider a model based on risk-equity constraints, while minimizing a global Value-at-Risk function.

### 3.4. Types of Algorithms (Computational Methods) Used

From a computational perspective, most of the existing models solve either a shortest path or a vehicle routing problem (VRP). A pure shortest path problem is usually trivial to solve with Dijkstra’s, label-setting, or label-correcting algorithms, and therefore we will not discuss those in much detail. On the other hand, VRPs are often very computationally demanding, and therefore often require a heuristic algorithm to solve.

As discussed earlier, multi-objective problems are usually represented as series of single-objective [40,45,51] or using several bi-objective problems [61]. Another general approach that has been used in several papers considers a two-stage framework; the inner subproblem solves for a shortest path exactly, while the outer master problem iterates VRP solutions [19,20,56]. It is also common to integrate exact and heuristic algorithms. For example, one could use an exact algorithm to find the shortest path, then apply a heuristic algorithm to find non-dominant solutions satisfying the objectives efficiently [40,45,46,51,64]. From a conceptual perspective, the literature can also be divided based on the focus of either (a) model development for a specific problem (authors compare different models for benchmarking), or (b) improving existent algorithms for obtaining solutions (benchmarking is achieved in terms of comparing the speed and whether an optimal solution is achieved). We present a tabulated summary of the algorithms used in the literature in Table 5.

## 4. An Example Integrating Predictive and Prescriptive Models

In this section, we use a simulated example to illustrate how different statistical/machine-learning risk models can impact the outcomes obtained from the prescriptive optimization models. The procedure for this example is comprised of three sequential steps. First, we use the Poisson distribution to simulate the number of crashes ($Y_{y}$) observed during any given trip. The rate of crashes is set to be a function of both precipitation and road traffic conditions whose distributions are assumed to be known. Given the simulated nature of the example, it allows us to know/compute the “true risk” associated with any trip. Then, in the second step, we use four popular predictive models (logistic regression, Poisson regression, neural networks, and XGBoost) to predict the probability of a crash or the number of crashes as a function of the aforementioned predictors. In the third step, we use the *k-shortest path* algorithm to identify the shortest routes ranked by the distance between two nodes [69]. Then, we conclude the third step by comparing the risk obtained as a result of the *k*-shortest path algorithm using each of the four crash risk predictive models.

### 4.1. Data Generation

We assume that the number of crashes, $Y_{i}$, can be generated from the following Poisson process,

(1) $$\begin{array}{cc} Y_{i} & ∼\mathrm{Poisson}(d_{i}\lambda_{i})\\ \log(\lambda_{i}) & =\beta_{0}+\beta_{1}x_{1i}+\beta_{2}x_{2i}+\epsilon_{i}\\ \epsilon_{i} & ∼\mathrm{Normal}(0,2^{2}), \end{array}$$

where $d_{i}$, $x_{1}$, and $x_{2}$ represent the *i*-th trip’s distance, precipitation, and traffic conditions, respectively. Note that (a) we have added a normally distributed random error as a noise term, and (b) the distance of each path $d_{i}$ is considered as the offset term in the Poisson distribution. We have arbitrarily set the following parameters,

(2) $$\begin{array}{cc} \beta_{0} & =-3,\;\beta_{1}=0.5,\;\beta_{2}=0.9,\\ d & ∼\mathrm{Poisson}(1000),\\ x_{1} & ∼\mathrm{Bernoulli}(0.15),\\ x_{2} & ∼\mathrm{Beta}(2,2). \end{array}$$

These parameters have been chosen to make the number of crashes $Y_{i}$ in all the simulated trips fall in a somewhat sensible range of 0 to 5. We have simulated 10,000 trips with various lengths under random precipitation and traffic condition to assess the performance of the four different predictive-prescriptive model combinations. The reader should note that the “true” risk is computed via the data generating process defined in Equation (1). To allow readers to replicate our analysis, we provide all the Python code used to simulate the data sets in the provided link in the Supplementary Materials.

### 4.2. Predictive Modeling

As an illustrative example, we have applied two traditional statistical models (logistic regression and Poisson regression) and two machine learning models (neural networks and XGBoost) to model crash risk in the simulated 10,000 trips. In the case of the Poisson regression approach, the outcome variable corresponds to the number of crashes (or more generally safety critical events such as hard brakes) in the path. On the other hand, the outcome variable for the other three models is binary, which indicates whether at least one event/crash has occurred. Thus, they can be considered as a simplification of the Poisson model implementation, where a practitioner would be interested in modeling the number of unsafe events instead of whether or not they occur. As the four models are predicting different outcomes, we have used the predicted rank of risk in each model to compare the concordance of prediction among the four models.

**Figure 1:**
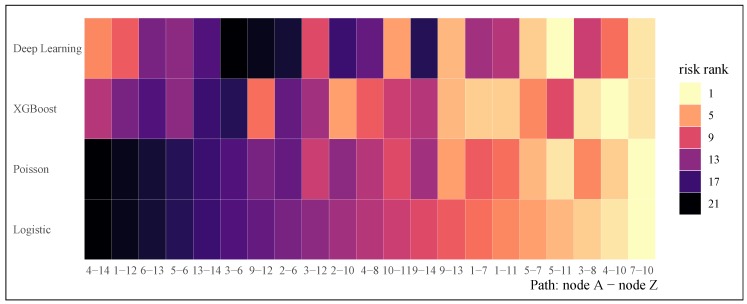
Concordance of four models for evaluating the risk of crash. Darker color indicates higher crash risk.

Figure 1 presents the concordance results with the logistic regression model used as a benchmark. As risk rank goes higher (the color of the tiles gets darker), the risk of events rises. The results show a higher concordance of prediction among the statistical models as well as among the machine learning models. There is less concordance across the statistical and machine learning models, for example, the highest risk paths (4–14, 1–12, and 6–13) predicted by statistical models are predicted to rank between 10 and 15 for the machine learning models.

Table 6 presents the model performance metrics for the four models. The difference of area under curve (AUC) between training and test set indicates that machine learning models have a minor issue of overfitting, which is commonly seen among machine learning models and requires state-of-art hyperparameter tuning and regularization. Neural networks in this case have very similar performance to logistic regression regarding accuracy and mean square error (MSE), but the AUC of test set is not as good as that of training set. Although the Poisson regression has the highest MSE, it does not indicate the Poisson regression has worse prediction than the other three models since the outcome variable is non-binary in this case. Among the three binary prediction models, logistic regression seems to have the best performance given the balance of performance between training and test set, as well as high AUC, accuracy, and low MSE. The reader should note that the four models were trained and measured using the h2o package in Python [70], and the concordance plot was generated using ggplot2 in **R** [71].

### 4.3. Prescriptive Modeling Using the *k*-Shortest Path Routing Algorithm

**Figure 2:**
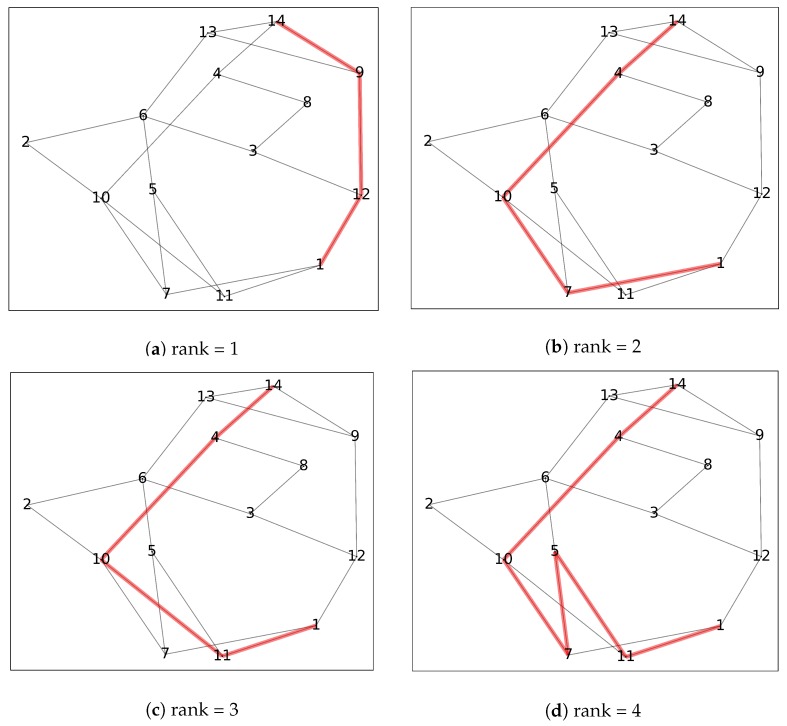
The results of the *k*-shortest path algorithm.

Here, we consider a road network including 14 nodes and 21 arches. Similarly, the weather and traffic conditions have been simulated using the same data generating process showed in Equation (2). With the help of k=4 shortest path algorithm, we find the four shortest paths from node 1 to node 14 and rank them by the corresponding distance. Figure 2 shows the selected path from rank 1 to 4. Furthermore, the rank of risk for each of those four paths using the four predictive models is provided in Table 7.

From Table 7, there are two observations that can be made. First, with the exception of neural networks, the rank of risk corresponds to the distance traveled. This indicates that the logistic regression, Poisson regression, and XGBoost models indicate that the shorter the route, the less likely one is involved in a crash. This is similar to the general assumption made by the majority of the optimization literature, where the crash probability is assumed to be a constant value of the distance traveled. On the other hand, the neural network shows an inverse relationship where for this simulated dataset, there may be some “safety” benefits from selecting longer routes. If one were to deploy the neural network model, in such a case, practitioners would need to balance the “cost” between risk and distance traveled. Second, the differences in crash risk ranking among the binary prediction models that have relatively similar performance predictive performances and the same selected features indicates that it is important to consider the effect of deploying these models on prescriptive models for decision-making. One can easily assume that, if the overall performance of the models are similar, the choice of implementing a given model would be similar. However, this example clearly shows that a closer examination/diagnosis of the predictive performance of these models is needed. For example, can we characterize the instances for which model is accurate? Note that, due to the simulated nature of this example, we do not discuss this issue further. The interested reader is referred to our Supplementary Materials for further analysis.

## 5. Conclusions

This review considers the prescriptive modeling aspects of data analytics approaches to improving motor vehicle safety. Specifically, we concentrate on optimization and operations research methods for routing, driver break scheduling, driver assignment, etc. The most significant conclusion permeating the review is the observation that there exists a gap between the conclusions of modern descriptive/predictive studies and the assumptions regularly made in the operations research models. We can observe that most authors agree that traffic risk significantly depends on uncertain and dynamically changing factors such as weather, traffic, driver status, etc. At the same time, most optimization models by design are not constructed to allow for either of these aspects (see Table 2).

It is also worth noting that, another piece sometimes missing from the literature is a thorough discussion of the value proposition of such techniques. Although intuitively it is clear that a reduction in traffic crash risks would be extremely beneficial to drivers, companies, and the society, in general, the existing literature (outside of hazmat applications) does not necessarily adequately measure the potential improvement or discuss the trade-off between safety and delivery efficiency. In the case of hazmat transportation, it is clear that ignoring crash risks can lead to catastrophic consequences, and the *exposed population* represents a key decision-making parameter. This parameter is relatively easy to measure, and consequently translate it into the operators’ liability. This then leads to clear advantages associated with using intelligent routing and scheduling. On the contrary, in non-hazmat cases, although it is possible to demonstrate statistically significant increase in crash risks associated with different conditions, this effect is not always large. For example, it is well demonstrated that texting while driving leads to a drastic increase in accident risk, leading to widespread adoption of corresponding laws and regulations. At the same time, there are not sufficient studies convincingly establishing that, for example, a dynamic routing policy that avoids severe weather conditions, reliably leads to a measurable improvement in driving safety. Partially, this is due to lack of practical implementations of safety-conscious routing in regular (non-hazmat) operations that takes advantage of the most recent developments in statistical crash prediction literature. At the same time, we cannot expect to see practical implementations until the value of such techniques is established more clearly.

Our main conclusion based on the reviewed literature is that the field is mature enough to produce a general-purpose safety-conscious routing engine for motor vehicle operators. Such an engine should be based on: (a) real-time feeds of weather and traffic data and forecasts, (b) pre-trained statistical models that evaluate driving conditions ahead, and (c) a collection of dynamic routing algorithms prescribing changes in the route as the conditions change. Each of these aspects individually has received a significant amount of attention in the respective research community. From our perspective then, there exists a distinct opportunity for data analytics to significantly contribute to motor vehicle safety, as long as the following issues related to merging results from these distinct streams of research are addressed.

(A) We have repeatedly observed the disconnect between the predictive and prescriptive models used in the literature. In our view, this is the most important gap in the literature. Before a practical implementation of safety-enabled dynamic routing for mainstream transportation can be achieved, a considerable effort in establishing best practices and guidelines is required. These efforts should primarily originate in the operations research community and should take advantage of the best ideas from the point above.
(B) In the absence of advanced dynamic routing models, it is difficult to adequately evaluate potential benefits of such systems. At the same time, the uncertainty in such an evaluation is a significant factor discouraging efforts in this area. We believe that a thorough analysis of the extent of potential risk-reduction with intelligent routing represents a primary research goal for the near future.
(C) The integration of risk prediction models with intelligent and dynamic routing models should be done with due diligence. As we showed in our simple simulation, an overall similarity in predictive performance does not necessarily lead to agreement on crash risk for a given path/route under certain conditions. Thus, researchers and practitioners should also attempt to diagnose/understand cases when the crash risk prediction models are performing poorly. Although this is more of a research-to-practice issue, we highlight this here to emphasize the possible dangers from deploying predictive models when their performance is not fully understood/analyzed.

## Figures and Tables

**Table 1:** An overview of hazmat risk models, their indicators, formulations, and application problems.

Model	Risk Indicator	Formula	Example Application Papers
TR	Traditional risk	$\min_{l\in P}\sum_{\left(i,j\right)\in A^{l}}p_{ij}Cij$	[40,44,45,46,47,48]
PE	Incident consequence	$\min_{l\in P}\sum_{\left(i,j\right)\in A^{l}}Cij$	[14,34,42,49]
IP	Incident probability	$\min_{l\in P}\sum_{\left(i,j\right)\in A^{l}}p_{ij}$	[43]
PR	Perceived risk	$\min_{l\in P}\sum_{\left(i,j\right)\in A^{l}}p_{ij}{\left(Cij\right)}^{k}$	[50,51]
MV	Mean-variance	$\min_{l\in P}\sum_{\left(i,j\right)\in A^{l}}\left(p_{ij}Cij+kp_{ij}{\left(C_{ij}\right)}^{2}\right)$	[52]
DU	Disutility	$\min_{l\in P}\sum_{\left(i,j\right)\in A^{l}}(p_{ij}\left(\exp\left(kC_{ij}-1\right)\right)$	[52]
MM	Maximum risk	$\min_{l\in P}\max_{\left(i,j\right)\in A^{l}}C_{ij}$	[52]
$MM_{2}$	MM (Uncertain probabilities)	$\min_{w}\max_{p}\sum_{\left(i,j\right)\in A^{l}}w_{ij}\left(p_{ij}C_{ij}+c_{ij}\right)$	[53]
CR	Conditional probability	$\min_{l\in P}\frac{\sum_{\left(i,j\right)\in A^{l}}p_{ij}C_{ij}}{\sum_{\left(i,j\right)\in A^{l}}p_{ij}}$	[54,55]
VaR	Value at risk (potential loss)	$\min_{\beta }P(R^{l}>\beta )\leq 1-\alpha$	[19,20,25]
CVaR	Conditional value at risk (Probability with large loss)	$\min E\{R^{l}|R^{l}\geq VaR_{\alpha }\left(R^{l}\right)\}$	[19,56]

**Notation:**$C_{ij}$ is the incident’s consequences; $P_{ij}$ is incident probability; *k* is risk preference parameter; α denotes the level of the confidence interval; β is the risk level; *A* reflects the set of arcs and i,j are used to represent each arc in *A*; *P* represents the set of different paths; and *l* denotes each path within *P*.

**Table 2:** An updated taxonomy of (hazmat) trucking optimization methods that consider crash risk/probabilities.

	Semi-Deterministic Models	Stochastic Models
**Truly-static**	**G1 Def.:** Risk only depends on the arc’s length and the binary variable for each arc denoting path selection. All the parameters considered are **deterministic** and the optimal solution does **not update**. **Examples:** [12,42,49,55,58,59,60,61]	**G2 Def.:** Risk only depends on the arc’s length and the binary variable for each arc denoting path selection. Model has ≥1 **random parameter(s)** and the optimal solution does **not update**. **Note:** This group cannot exist in practice since the inclusion of a random parameter will make the optimal solution changeable according to the conditions.
**Semi-dynamic**	**G3 Def.:** Risk only depends on the arc’s length and the binary variable for each arc denoting path selection. All other parameters are fixed. The optimal solution is a **conditional decision**, which will be different according to the realization of parameters. **Examples:** [20,25,56]	**G4 Def.:** Risk depends only on the arc’s length and the binary variable for each arc denoting path selection. Model has ≥1 **random parameter(s)**. The optimal solution is a **conditional decision**, which will be different according to the realization of parameters and value of stochastic input(s). **Examples:** [14,19,40,42,45,46,47,48,51,62,63,64,65,66]
**Truly-dynamic**	**G5 Def.:** Risk only depends on the arc’s length and the binary variable for each arc denoting path selection. Other parameters are fixed. **The model has criteria to update the solution** (i.e., run the model based on querying the values of parameters) in real-time. **Examples:** None found.	**G6 Def.:** Risk depends only on the arc’s length and the binary variable for each arc denoting path selection. Model has ≥1 **random parameter(s)**. **The model has criteria to update the solution** (i.e., run the model based on querying the values of parameters) in real-time. **Examples:** [67]

**Table 3:** Type of input parameters included within trucking safety oriented optimization models.

Type ID	Type of Parameter	Example Papers and Applications
1	Risk parameters including probability of accident and/or expected consequence	These parameters are included in all safety-based routing optimization papers and thus, we will not highlight specific papers here
2	Parameters for the traditional vehicle routing problem (VRP)	[40,45,46,47,48,51,60,64]
3	Parameters about the confidence interval of accident or the worst case	[19,56,63]
4	Parameters of travel time	[14,19,45,46,51,64,67]
5	Parameters about traffic condition	[56,63,65]
6	Parameters about weather condition	[58,65,67]
7	Parameters of dispersion model to calculate the concentration level	[58,62,64]
8	Parameters about road geometric condition	[65,67]
9	Parameters about traveling cost	[60,65]
10	Parameters about the threshold of accident probability or/and consequence	[55]
11	Parameters about equity constraint	[25]

**Table 4:** Details about objective function(s) and parameter type ID (PT-ID) used in the literature.

Objective	Details about Objective in Model	Papers	PT-ID
Minimize cumulative VaR for all hazmat routes	VaR is used in these two papers to denote the maximum cutoff risk for each arc due to hazmat transportation	[25]	1, 3, 11
		[20]	1, 3
	VaR denotes the risk level, such that the risk for each selected arc exceeding a certain risk level is ≤ a pre-specified probability threshold	[56]	1, 3
Minimize CVaR	CVaR is a coherent risk measure to avoid ignoring low-probability highly consequential crashes	[56]	1, 3
		[19]	1, 3, 4
Minimize travel cost and/or risk	Population exposure and travel time	[14]	1, 4
	Travel cost and risk exposure costs such as population exposure, facilities-related exposure, and pavement-related exposure	[60]	1, 2, 9
	Traditional risk (the product of risk probability and the consequence) and travel time	[47]	1, 2
		[64]	1, 2, 4
		[40]	1, 2
		[45]	1, 2, 4
	Perceived risk (PR) and travel time	[51]	1, 2, 4
	Direct travel cost and the risk cost depends on frequency of risk and leakage probability	[65]	1, 5, 6, 8, 9
	Total risk, which is defined in this application as the total expected concentration level of gas or aerosols when an accident happens	[58]	1, 7
	Population Exposure model (including travelers)	[42]	1, 5
	Conditional expectation of the consequence given an accident happens (at the same time the probability of accident for the path cannot exceed a certain number and also the consequence should lower than or equal to a threshold)	[55]	1, 10
	Total number of vehicles, scheduling time, and the traditional risk (TR)	[46]	1, 2, 4

**Table 5:** Algorithms used in the mathematical/optimization models accounting for crash risk.

Type	Description of the Algorithm	Example Papers
**Exact**	Branch-and-Bound	[55,68]
	Branch-and-Bound with a relaxing risk equity constraint as the penalty parameter in the objective function	[25]
	*Two-stage solution:* Inner stage is to the solve shortest path problem using Dijkstra’s algorithm; Outer loop is an algorithm to select a solution to minimize VaR and CVaR	[20,56]
	*Two-stage solution:* Sub-problem uses a back-labeling algorithm to solve the dynamic shortest path problem; Main problem is a CVaR minimization problem by the proposed algorithm	[19]
	An approach using STDLT(DD), STDLT(SD) and EV algorithms	[14]
**Heuristic**	An insertion heuristic algorithm is used to determine non-dominated scheduled route-paths; then a newly proposed label setting algorithm is used to identify the entire set of k-shortest scheduled route-paths	[51]
	Based on the shortest path algorithm, the bi-objective VRP is decomposed to single objective problems, then solved using an insertion heuristic algorithm to approximate a set of non-dominated solutions	[40,45]
	Multiple objectives are converted to a bi-objective problem using a decomposition method; then a proposed constrained parametric method is applied to solve the shortest path problem and transfer the bi-objective problem to two single objectives	[61]
	A labeling algorithm is applied to find the shortest path between customers and the depot, then a MOACS-based algorithm is used to find a set of non-dominant solutions for the VRPTW	[47]
	An algorithm based on a heuristic GA is applied to solve HVRPTW	[60]
	A route-building heuristic algorithm based on a label-setting algorithm is used to solve the single objective time-dependent shortest path problem	[64]
	Meta-heuristic algorithm based on an ACS is supported by labeling algorithm for HVRPTW	[46]

**Acronyms:** STDLT: Stochastic, Time-Dependent Least Time; DD: Deterministic Dominance; SD: Stochastic Dominance; EV: Expected Value; VRP: Vehicle Routing Problem; MOACS: Multiple Objectives Ant Colony System; VRPTW: Vehicle Routing Problem with Time Windows; GA: Genetic Algorithm; HVRPTW: Hazmat Vehicle Routing Problem with Time Windows; ACS: Ant Colony System.

**Table 6:** Performance metrics for the four predictive models.

Model Performance Metrics	Logistic	Poisson	XGBoost	Neural Networks
train AUC ${}^{1}$	0.5596	—	0.6024	0.5743
test AUC ${}^{1}$	0.5639	—	0.5456	0.5327
train accuracy	0.8936	—	0.8933	0.8936
test accuracy	0.8941	—	0.8941	0.8941
train MSE ${}^{2}$	0.0948	0.1647	0.2353	0.0949
test MSE ${}^{2}$	0.0938	0.1717	0.2352	0.0954

${}^{1}$ Area Under Curve (AUC) ranges between 0.5 and 1. Higher values suggest better models. ${}^{2}$ Mean Square Error (MSE) is a positive number. Smaller values suggest better models

**Table 7:** Risk ranking for the k→=4 shortest paths using the four predictive models.

Path	Rank by Distance	Rank by Logistic Regression	Rank by Poisson Regression	Rank by XGBoost	Rank by Neural Networks
1→12→9→14	1	1	1	1	4
1→7→10→4→14	2	2	2	2	3
1→11→10→4→14	3	3	3	3	2
1→11→5→7→10→4→14	4	4	4	4	1

## Supplementary Materials

In an effort to bridge the gap between the crash prediction literature and the hazmat/optimization literature, we have made all our source code for (a) simulating data, (b) crash risk modeling using the four statistical and machine learning algorithms, (c) visualizing crash ranks predicted by the four models, and (d) *k*-shortest path routing, available on the following GitHub repository: https://github.com/caimiao0714/optimization_stats_case_study. The code is provided in an interactive Jupyter Notebook to allow the reader to simultaneously view both the code and the corresponding results/analysis.

## Abbreviations

The following abbreviations are used in this manuscript:

AUC	Area Under Curve
Hazmat	Hazardous Materials
MDP	Markov Chain Process
MSE	Mean Square Error
O–D	Origin-Destination
VRP	Vehicle Routing Problem
